# Supplementary material for: Integrated dual-laser photonic chip for high-purity carrier generation enabling ultrafast terahertz wireless communications
Source: Nat Commun. 2022 Mar 16;13:1388. doi: 10.1038/s41467-022-29049-2 (PMC8927353; doi:10.1038/s41467-022-29049-2)
Supplement: Supplementary file 1 — Supplementary Information [file 41467_2022_29049_MOESM1_ESM.pdf]

Supplementary Information

Integrated Dual-Laser Photonic Chip for High-Purity Carrier Generation Enabling Ultrafast Terahertz Wireless Communications

Shi Jia[1,9], Mu-Chieh Lo[2,7,9], Lu Zhang[3,5,9], Oskars Ozolins[3,4,8], Aleksejs Udalcovs[4], Deming Kong[1], Xiaodan Pang[3*], Robinson Guzman[2], Xianbin Yu[5], Shilin Xiao[6], Sergei Popov[3], Jiajia Chen[3], Guillermo Carpintero[2*], Toshio Morioka[1], Hao Hu[1*], and Leif K. Oxenløwe[1]*

*[1]DTU Fotonik, Technical University of Denmark, DK-2800, Kgs. Lyngby, Denmark. [2]Universidad Carlos III de Madrid, 28911 Leganés, Madrid, Spain. [3]KTH Royal Institute of Technology, 164 40 Kista, Sweden. [4]RISE Research Institutes of Sweden, 164 40 Kista, Sweden. [5]College of Information Science and Electrical Engineering, Zhejiang University, Hangzhou 310027, China. [6]School of SE-IEE, Shanghai Jiao Tong University, Shanghai 200240, China. 7 Optical Networks Group , University College London, London WC1E 7JE, United Kingdom. [8]Institute of Telecommunications, Riga Technical University, Riga LV-1048, Latvia. [9]These authors contributed equally: Shi Jia, Mu-Chieh Lo, Lu Zhang. *Corresponding Authors, email: shijai@fotonik.dtu.dk, xiaodan@kth.se, guiller@ing.uc3m.es and huhao@fotonik.dtu.dk.*

Supplementary Information.

1) Design of the monolithically integrated dual-DFB lasers chip

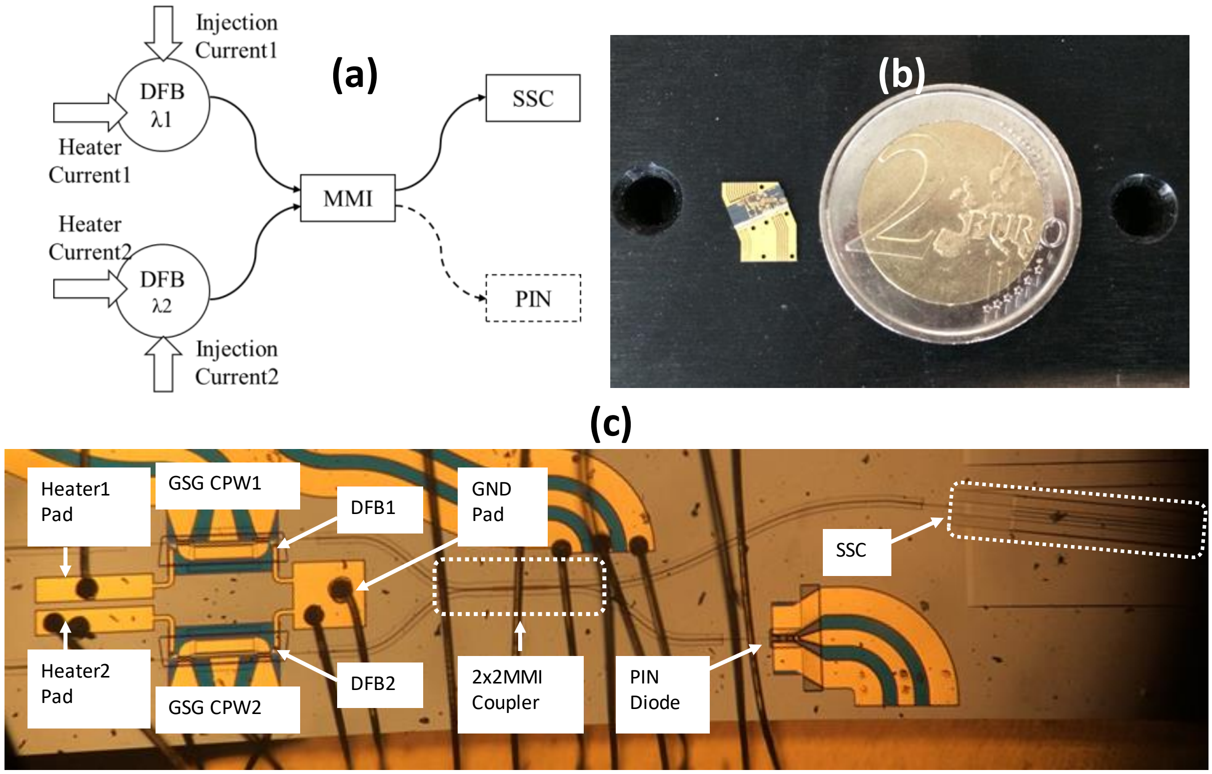

Supplementary Fig. 1(a) Schematic of the PIC. (b) Top view of the PIC placed next to a 2-euro coin. (c) Microscopic view of the PIC.

Supplementary Fig. 1(a) depicts the schematic of the PIC. Two DFB lasers are placed parallel and combined through the MMI coupler prior to the SSC (optical output) and the PIN (electrical output). Each DFB laser is controlled with heater current and injection current. Supplementary Fig. 1(b) presents the photograph of the PIC, and Supplementary Fig. 1(c) presents the microscopic view of the PIC. In Supplementary Fig. 1(c), two DFB lasers are placed in the upper-left and lower-left, respectively. The default emission wavelengths are set to 1550 nm for DFB1 and 1554 nm for DFB2 by properly defining the gratings. Each standardized DFB laser building block contains a GSG CPW along with an internal heater for thermo-optic tuning. The DC ports for the heater pad

and ground (GND) pad can be connected to freely routed metal tracks. The ground pad ports of two DFB lasers are shorted with DC routing and one GND pad for simplified wire bonding. The GSG CPWs support RF signal injection, which is not used in this paper. Injection currents are injected onto the GSG CPWs, and heater currents are fed onto the heater pads. Driven with these bias currents, the intensity and wavelength of the two DFB lasers are precisely controlled. With the butt-joint active-passive transitions and deeply etched passive waveguide sine bends, the ridge-waveguide stripe DFB lasers are coupled to the following deeply etched 2x2 MMI coupler. The optical signals the two DFB lasers emit are combined through the MMI coupler. After the MMI coupler, one split output signal travels along the upper path, another transition and sine bend towards the 7° angled spot-size converter (SSC). The SSC adiabatically tapers the shallowly etched waveguide mode to a size that gives maximum overlap and minimum distortion. Besides maximizing the mode overlap efficiency, the SSC guides the combined light out of the chip on the anti-reflection (AR) coated front facet for optimized fiber coupling. The other split output waveguide of the MMI coupler is routed to the PIN photodiode. However, the PIN photodiode supports up to 40 GHz, far below the frequency of interest for THz communications, and not used in this paper. The dimension of the PIC is 4.5mm x 1 mm, as shown in Supplementary Fig. 1(b). The 250-μm-thick InP chip is mounted on a 0.4-mm-thick submount and placed on a copper block with an active Peltier thermoelectric cooler (TEC) on the surface and controlled with a TEC controller.

2) The details of digital signal processing (DSP) and optimization of DSP parameters

At the receiver, the signal is first captured from the scope, and then we use the autocorrelation of pseudo-noise (PN) sequence to find the frame head in the captured frame to perform synchronization. The autocorrelation results of the signal captured from the scope are shown in

Supplementary Fig. 2. It can be seen that the synchronization head-to-noise ratio is more than 25dB. Thus, the PN sequence-based synchronization method is efficient in the THz OFDM communication system.

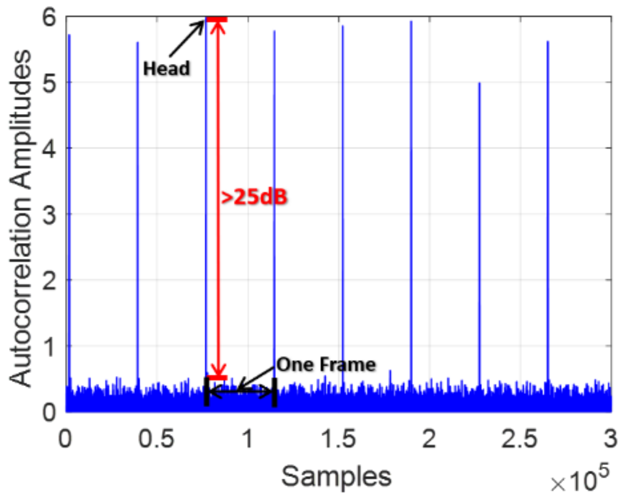

Supplementary Fig. 2 Synchronization of THz OFDM frame.

After synchronization, the CP is first removed to mitigate the influence of multi-path interference induced ISI. Then, the fast Fourier transform (FFT) module transforms the time domain symbols into the frequency domain to realize the OFDM demodulation. After that, equalization is used to recover back the signal. The channel equalization is composed of linear equalization (LE), nonlinear equalization (NE), and phase noise compensation (PNC).

First, the signal after the FFT module passes through the pilot-based one-tap LE, used to compensate for the linear response of the system. We assume that $P(m,l)$ represents the pilot carried with subcarrier index m at the i-th symbol, $P'(m,l)$ represents the corresponding received pilot. The number of pilots is N_p, and the estimation of the linear channel response at the m-th subcarrier is expressed as:

$$H(m)=\left(\sum_{l=1}^{N_p} P'(m,l)/P(m,l)\right)/N_p\,. \quad (1)$$

The number of the total OFDM symbols is denoted as N_s, and the index l of the pilots corresponds

to:

$$l = (k-1) \cdot N_s / N_p + 1, \quad k = 1, ..., N_p \,. \tag{2}$$

After LE, the signal is equalized with $H(m)$. Assuming that $R(m, l)$ represents the received data with subcarrier index m at the i-th symbol, the data $Y(m,l)$ after LE is expressed as:

$$Y(m,l) = R(m,l) / H(m) \,. \tag{3}$$

$Y(m, l)$ is first re-modulated to OFDM signal, which flows the same routine of the transmitter, and then equalized with a PNC. Assume $y'(n)$ is the re-modulated signal, then the phase noise is calculated according to Eq. (4).

$$\theta = decision\{arg(y(n) / y'(n))\} \,, \tag{4}$$

where $arg(.)$ means the angle of the variable, $decision\{.\}$ means the filtering operation to filter out the high-frequency distortions in the phase noise. After that, the PNC is loaded to the received signal $y(n)$, which yields:

$$y_2(n) = e^{j\theta} \cdot y(n) \,, \tag{5}$$

The BER versus the training overhead with and without PNC is shown in Supplementary Fig. 3. With the increase of LE training overhead, the BER with PNC and without PNC improved gradually. There are significant system performance improvements after PNC. Finally, we choose 10% training overhead in the experimental setup, and it has been extracted in the calculation of system data rate.

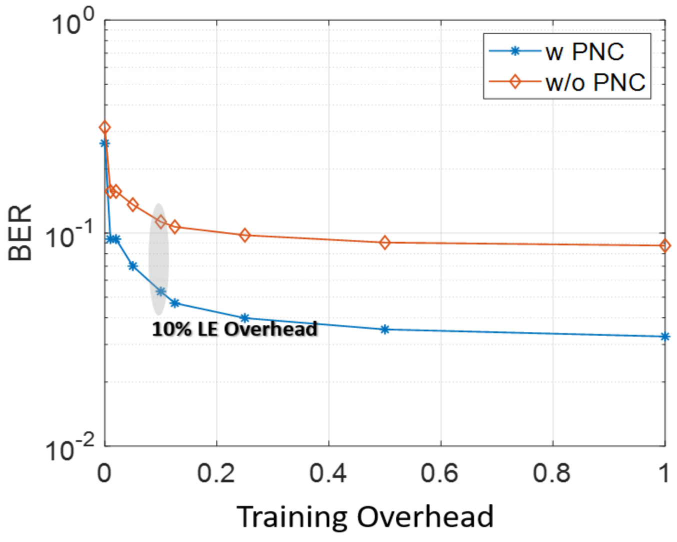

Supplementary Fig. 3 BER versus the LE training overhead.

After PNC, the Volterra series nonlinear model is used for estimating the nonlinearity components, which considers only the 2nd-order and partially the 3rd-order terms. In the time domain, the transmission model is expressed as follows:

$$\begin{aligned} y_2(n) = {} & w + \\ & \sum_{i=0}^{M_1-1} h_1(i)x(n-i) + \\ & \sum_{i=0}^{M_2-1}\sum_{j=i}^{M_2-1} h_2(i,j)x(n-i)x(n-j) + \\ & \sum_{i=0}^{M_3-1}\sum_{j=i}^{M_3-1}\sum_{k=j}^{M_3-1} h_3(i,j,k)x(n-i)x(n-j)x(n-k) + \ldots\ldots \end{aligned} \quad (6)$$

where n is the index of time-domain samples, $x(n)$ is the transmitted OFDM signal, w is the additive Gaussian noise originating from the device background noise and channel impairments, $h_K(\ldots)$ (K>0) is the K-th order Volterra kernel corresponding to the linear (K=1) or nonlinear (K>1) channel responses, and M_K is the memory length of K-th order effect. Since linear distortions (w and h_1) have been well compensated with low complexity and low overhead LE in Eq. (3), we only consider h_2 and h_3 in Eq. (6) for nonlinear compensation. The nonlinear kernels are estimated by a recursive least square (RLS) algorithm using the training symbols, which is an iterative process

shown as follows:

$$\boldsymbol{e}(\boldsymbol{i}) = \boldsymbol{y}_2^{\boldsymbol{i}}(\boldsymbol{n}) - \tilde{\boldsymbol{x}}^{\boldsymbol{i}}(\boldsymbol{n})^{\boldsymbol{T}}\hat{\boldsymbol{h}}^{\boldsymbol{i}\text{-}1}(\boldsymbol{n}), \tag{7}$$

$$\boldsymbol{k}^{\boldsymbol{i}}(\boldsymbol{n}) = \mathrm{P}^{\boldsymbol{i}-1}(\boldsymbol{n})\tilde{\mathrm{x}}^{\boldsymbol{i}}(\boldsymbol{n})\{\boldsymbol{\lambda} + \tilde{\mathrm{x}}^{\boldsymbol{i}}(\boldsymbol{n})^{\boldsymbol{T}}\mathrm{P}^{\boldsymbol{i}\text{-}1}(\boldsymbol{n})\tilde{\mathrm{x}}^{\boldsymbol{i}}(\boldsymbol{n})\}^{-1}, \tag{8}$$

$$\mathrm{P}^{\boldsymbol{i}}(\boldsymbol{n}) = \boldsymbol{\lambda}^{-1}\{\mathrm{P}^{\boldsymbol{i}\text{-}1}(\boldsymbol{n}) - \boldsymbol{k}^{\boldsymbol{i}}(\boldsymbol{n})\tilde{\mathrm{x}}^{\boldsymbol{i}}(\boldsymbol{n})^{\boldsymbol{T}}\mathrm{P}^{\boldsymbol{i}\text{-}1}(\boldsymbol{n})\}, \tag{9}$$

$$\hat{\boldsymbol{h}}^{\boldsymbol{i}}(\boldsymbol{n}) = \hat{\boldsymbol{h}}^{\boldsymbol{i}\text{-}1}(\boldsymbol{n}) + \boldsymbol{e}^{\boldsymbol{i}}(\boldsymbol{n})\boldsymbol{k}^{\boldsymbol{i}}(\boldsymbol{n}). \tag{10}$$

In the i-th iteration, the estimated $\hat{h}$ is updated by Eq. (10) with the error signal e^i in Eq. (7) and the gain vector k^i from Eq. (8) and Eq. (9). In Eqs. (7)-(10), the index n denotes the n-th subcarrier, λ is a predefined constant before initialization, set as 0.9999 in our experiment. P(n) is the inverse correlation matrix of the input signal. After obtaining the nonlinear kernels with the RLS algorithm and training symbols, the signal with nonlinear distortions is equalized by subtracting the nonlinear noise using Eq. (11) with estimated h'.

$$y'(n) = y_2(n) - \sum_{i=0}^{M_2-1}\sum_{j=i}^{M_2-1} h_2'(i,j)x'(n-i)x'(n-j) - \sum_{i=0}^{M_3-1}\sum_{j=i}^{M_3-1}\sum_{k=j}^{M_3-1} h_3'(i,j,k)x'(n-i)x'(n-j)x'(n-k). \tag{11}$$

After equalization with Eq. (11), $y'(n)$ is utilized as the input for a decision-feedback module for the next iteration until the loop comes to the predefined iteration loop number.

The BER versus the memory length of Volterra filtering is shown in Supplementary Fig. 4. When nonlinear compensation is considered, the BER is greatly improved from 0.054 to low-density parity-check convolutional codes (LDPC-CC) forward error correction (FEC), and the number of OFDM symbols used to train the Volterra model does not influence much. Finally, we choose memory length equals 4 and 2 OFDM symbols to train the Volterra filtering in the experimental setup, and it has been extracted in the calculation of system data rate.

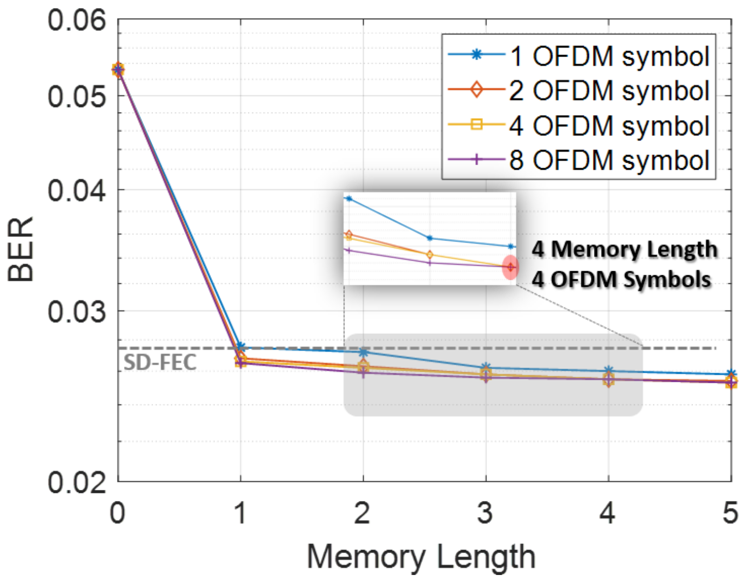

Supplementary Fig. 4 BER versus the memory length of Volterra filtering.

3) The details of the calculation process of the system net rate

In the experiment, the bandwidth of the 16-QAM-OFDM signal is 44.43 GHz. The gross rate of the system is calculated by the production of the signal bandwidth and the spectrum efficiency, which equals 177.72 $Gbits^{-1}$ (44.43 GHz×4 bits·Hz^{-1}). The CP, the LE pilot, and the FEC coding occupy the main part of the system overhead in the DSP routine, whereas the PNC and the NLE only need to be updated in the training stage. The length of the CP and IFFT are 16 and 1024, respectively, which corresponds to the overhead of 16/(16+1024)≈1.538%. The overhead of the LE pilot is 10%. Besides, the pseudo-code-based synchronization frame also occupies a negligible part of the transmitted frame since it only has 127 samples and has around 0.1% overhead. First, the system line rate is calculated without considering the FEC overhead, which equals 157.46 Gbit/s (177.72 $Gbits^{-1}$ ×(1-1.538%)×(1-10%)×(1-0.1%)). Then, the system transmission performance reaches the LDPC-CC FEC threshold (2.7E-2), which occupies 20%

overhead, and the system net rate is calculated extracting the FEC overhead, which equals 131.21 Gbits^{-1} (157.46 Gbit/s/(1+20%)).

4) Link Budget Calculation

The input optical power into the UTC-PD is +14 dBm, and the conversion efficiency is 0.15 AW^{-1}. After heterodyne detection in the UTC-PD, the power of the output THz signal is around -24 dBm. The free space path loss (FSPL) is 10*log(4*π*10.7*408e9/3e8)2≈105 dB. The total antenna gain of the two antenna/lens combinations is 103 dBi, which is estimated by comparing the received power with the calculated FSPL. The power of the received THz signal is -24+103-105=-26 dBm. The conversion loss of the THz mixer is around 17dB, and the gain of the intermediate frequency (IF) amplifier (SHF 807B) is 23 dB. Thus, the power of the IF signal is -26-17+23=-20 dBm. Then, the IF signal is captured by DSO for signal processing.

Considering the SNR calculation, the THz mixer and the IF amplifier dominate the noise contributions to the received signal. The average noise level of the THz mixer (VDI WR2.2MixAMC) is -151 dBm·Hz^{-1}. The noise power is 7.94e-19 W/Hz*44.43 GHz=3.53e-8 W=-44.5 dBm. The SNR of the IF signal before the IF amplifier is around -26-(-44.5)=18.5 dB. The noise figure (NF) of the IF amplifier is 4 dB, and the SNR of the IF signal after the IF amplifier is around 18.5-4=14.5 dB.

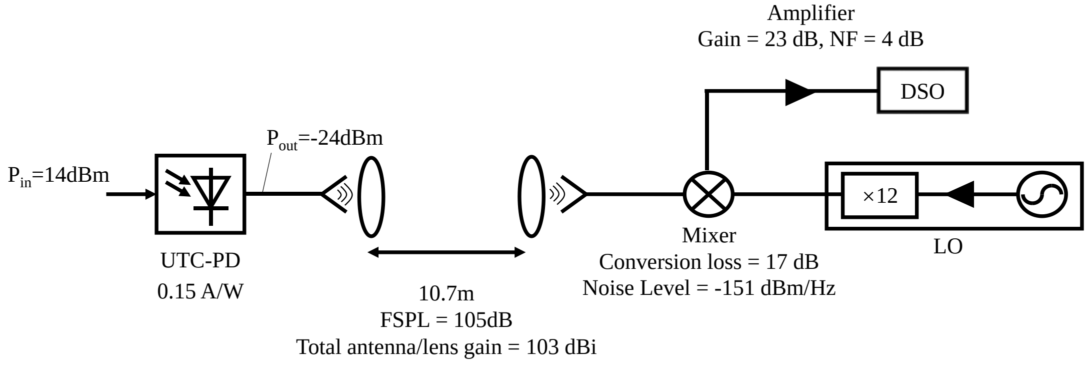

Supplementary Fig. 5 Link budget of the experiment system.
